# Unraveling the microbiological and chemical basis of aroma differences in Great Wall cigars: insights from amplicon sequencing and volatile metabolomics

**DOI:** 10.3389/fmicb.2026.1952964

**Published:** 2026-09-25

**Authors:** Xiang Dai, Mingxuan Yang, Yangyang Gao, Jinshan Lei, Keyuan Ye, Ting Zhou, Qi Chen, Yonghao Tan, Haoyu Yan, Lanxin Ma, Yumeng Zhang, Huina Zhou, Jun Hu, Xi Hu

**Affiliations:** 1 Key Laboratory in Flavor and Fragrance Basic Research, Zhengzhou Tobacco Research Institute, China National Tobacco Corporation, Zhengzhou, China; 2 Development & Industrial Efficient Utilization of Domestic Cigar Tobacco Key Laboratory of Sichuan Province, China Tobacco Sichuan Industrial Co., Ltd., Chengdu, China; 3 China Tobacco Sichuan Industrial Co., Ltd., Chengdu, China; 4 China Tobacco Gene Research Center, Zhengzhou Tobacco Research Institute, China National Tobacco Corporation, Zhengzhou, China

**Keywords:** amplicon sequencing, aroma, cigar, correlation analysis, microbial community, volatile metabolomics, volatile organic compounds

## Abstract

To clarify the microbiological and chemical bases underlying aroma differences among six Great Wall cigar types, 16S rRNA/ITS amplicon sequencing and headspace solid-phase microextraction coupled with gas chromatography–mass spectrometry (HS-SPME-GC-MS) were integrated to characterize microbial community composition, volatile organic compound (VOC) profiles, and potential microbe–VOC associations. Microbial diversity and community composition differed among the six cigar types, and linear discriminant analysis effect size (LEfSe) identified 16 potential microbial biomarkers. Volatile metabolomics analysis identified 1,620 VOCs, which were classified into nine chemical classes. The combined results of relative odor activity value (rOAV) analysis and partial least squares discriminant analysis (PLS-DA) revealed 17 VOCs as key aroma compounds associated with aroma differences among the six cigar types. Notably, representative compounds, including β-damascenone (floral, fruity, sweet), dihydro-2-methyl-3(2H)-furanone (roasted, nutty, sweet), δ-cadinene (herbal, woody), and δ-dodecalactone (creamy, fruity), exhibited distinct rOAV distribution patterns across the six cigar types, which may contribute to the characteristic aroma profiles of these cigars. Correlation analysis further revealed that several microbial genera were significantly associated with key aroma compounds. In particular, the bacterial genus *Atopostipes* and the fungal genus *Sampaiozyma* displayed significant positive correlations with dihydro-2-methyl-3(2H)-furanone, β-damascenone, and other key aroma compounds. These findings provide useful insights for screening aroma-producing microorganisms and improving cigar quality control.

## 1 Introduction

As high-quality tobacco products, cigars are renowned for their unique, complex, and well-balanced aroma profiles. Cigar production involves a series of postharvest processes, such as air-curing, fermentation, and aging, which collectively shape the aroma profiles of cigar products. As one of the major quality attributes, cigar aroma is closely associated with volatile organic compounds (VOCs), and variations in VOC profiles contribute substantially to the distinct sensory quality observed among different cigars (Zhou et al., 2024). In addition to VOCs, microbial communities are also recognized as important contributors to cigar aroma development (Xue et al., 2023). Specifically, certain microorganisms can secrete extracellular enzymes which degrade macromolecules such as lipids, starch, and proteins within the tobacco matrix. This enzymatic hydrolysis releases aroma precursors, including reducing sugars and amino acids. Subsequently, these precursors may be further transformed through Maillard reactions, oxidative degradation, microbial metabolism, and related esterification processes into diverse aroma components, including alcohols, terpenoids, aldehydes, esters, and heterocyclic compounds. Therefore, elucidating the associations between microbial communities and VOC profiles is necessary for understanding the formation of distinctive aroma profiles across different cigar types.

Recently, advances in volatile metabolomics and high-throughput sequencing have facilitated the comprehensive characterization of microbial communities and VOC profiles in cigars. For instance, Zhou et al. (2024) used sensory evaluation, headspace gas chromatography–ion mobility spectrometry (HS-GC-IMS), and electronic nose (E-nose) analysis to characterize the aromatic style of six cigar products. They detected 88 volatile compounds and further identified 24 compounds as key differential volatile components, including 2-heptanone, n-butanol, and 2,6-dimethylpyrazine. Furthermore, Xue et al. (2023) used metagenomic analysis to reveal that *Bacillus pumilus* and *Pseudomonas* sp. 286 were potential microorganisms involved in starch degradation, while *Corynespora cassiicola* and *Pseudomonas fulva* were associated with alkaloid degradation in cigars. However, these studies on cigars have generally investigated VOCs or microbial communities separately, thereby leaving the mechanisms responsible for cigar aroma formation poorly understood.

Given the complexity of cigar aroma formation, integrated multi-omics approaches have increasingly been employed to elucidate the mechanisms involved in this process. Specifically, Gao et al. (2025) integrated VOC profiling, metagenomics, and untargeted metabolomics to examine the impact of initial water content on the final quality of fermented cigar tobacco leaves. They found that predicted microbial functions associated with carbohydrate metabolism were significantly more abundant during the early and middle phases of fermentation under 20% water content. Moreover, Zhang et al. (2025) combined headspace solid-phase microextraction coupled with gas chromatography–mass spectrometry (HS-SPME-GC-MS) and amplicon sequencing to investigate the changes in volatile flavor compounds and microbial communities across the air-curing stages. Their results showed that compared with fungal communities, bacterial communities exhibited greater microbial diversity and richness and displayed stronger associations with volatile flavor compounds. Additionally, Wu et al. (2024) investigated dynamic changes in metabolite profiles and microbial communities during different mold stages in cigar tobacco leaves. They observed that mold contamination increased the relative content of nicotine but reduced the relative content of ketones and alcohols. Concurrently, the microbial co-occurrence network became less complex and less stable. Although multi-omics strategies have provided valuable insights into aroma formation in cigar tobacco leaves, integrated analyses linking microbial communities to VOC profiles across diverse cigar types are still rare.

Thus, this study systematically investigated the volatile profiles and microbial community composition among six Great Wall cigar types using HS-SPME-GC-MS and amplicon sequencing. We focused on identifying key aroma compounds and potential microbial biomarkers associated with aroma differences among the six cigar types, while exploring associations between these biomarkers and key aroma compounds. These findings deepen our understanding of the chemical and microbiological basis underlying cigar aroma characteristics and provide theoretical support for producing high-quality cigars.

## 2 Materials and methods

### 2.1 Sample preparation

Six Great Wall cigar types with distinctive aroma profiles were selected for this study: Cameroon International (S1), Flourishing Age V (S2), Yichuan Laofang (S3), Miracle 132 (S4), Spectacular No. 2 (S5), and VegaFina Grand Corona (S6). All cigars were supplied by the Great Wall Cigar Factory of China Tobacco Sichuan Industrial Co., Ltd. To ensure sample consistency and reduce manufacturing-related variation, the rolling process for all cigars was completed by the same skilled roller. After rolling, all cigars were immediately placed in a −20 °C freezer for 48 h to reduce the risk of insect infestation. Subsequently, the cigars were transferred to an aging cabinet (SICAO, C5, Foshan, China) and aged at 20°C ± 2 °C and 60 ± 2% relative humidity for 180 days. After aging, six individual cigars were randomly selected from each cigar type and used as independent biological replicates. Each selected cigar was processed separately: frozen in liquid nitrogen, ground to a fine powder, and then transferred into a separate sterile bag. The powder from each biological replicate was then separated into two aliquots, which were used for VOC analysis and microbial community sequencing, respectively.

### 2.2 Extraction and identification of VOCs

The VOCs of the cigar samples were characterized by HS-SPME-GC-MS using an Agilent 8890/7000D system, according to the procedure reported by Yang et al. (2025b). Briefly, 500 mg of the cigar sample was placed in a 20 mL headspace vial (Agilent, Palo Alto, CA, USA), followed by the addition of 2 mL of saturated NaCl solution and 10 μL of an internal standard solution (3-hexanone-2,2,4,4-d_4_, 10 μg/mL). After incubation with constant shaking at 60 °C for 300 s, the VOCs were extracted using a 120 μm DVB/CWR/PDMS SPME Arrow (Agilent, Palo Alto, CA, USA) at 60 °C for 15 min. After extraction, the SPME Arrow was immediately introduced into the GC injection port, where the extracted analytes were thermally desorbed at 250 °C for 300 s. GC-MS analysis was performed on an Agilent 8,890 gas chromatograph coupled to an Agilent 7000D triple quadrupole mass spectrometer (Agilent Technologies, Santa Clara, CA, USA). Chromatographic separation was achieved on a DB-5MS capillary column (30 m × 0.25 mm × 0.25 μm). Detailed chromatographic and mass spectrometric conditions are referred to our previous method (Yang et al., 2025b). To monitor instrumental stability throughout the analytical sequence, a pooled quality control (QC) sample, comprising equal portions of each sample, was injected after every ten sample analyses. The MWGC database (Yuan et al., 2022) was used as the reference library to identify the VOCs based on their mass spectra, retention times (RTs), and characteristic ions. Briefly, for each target compound, one quantitative ion and two to three qualitative ions were selected and monitored within the corresponding retention-time window. A compound was considered identified when its detected RT and all pre-selected characteristic ions were consistent with the corresponding MWGC database entry. Additionally, the relative content of each VOC was calculated by comparing its peak area with that of the internal standard. The calculation was performed using the following equation:


$$X_{i}=\frac{V_{s}*C_{s}}{M}*\frac{I_{i}}{I_{s}}$$

where C_s_ and V_s_ are the concentration (μg/μL) and volume (μL) of the added internal standard, respectively; M refers to the sample mass (0.5 g); I_i_ and I_s_ denote the peak areas of compound i and the internal standard, respectively.

### 2.3 Calculation of relative odor activity values (rOAVs)

The potential aroma contribution of VOCs in each cigar type was evaluated using rOAVs, which were calculated following the method described by Peng et al. (2024). Accordingly, VOCs were considered as aroma-active compounds when their rOAVs exceeded 1, whereas those with rOAVs ranging from 0.1 to 1 were classified as potential aroma contributors (Li et al., 2025). The rOAV of each target compound i was calculated as follows:


$$r\,O\,A\,V_{i}=\frac{X_{i}}{T_{i}}$$

where X_i_ and T_i_ denote the relative content and the odor threshold of the target compound i, respectively, with both values expressed in μg/g. Odor descriptors and odor threshold values were obtained from the Good Scents Company^1^, the Perflavory database^2^, the LRI & Odor Database–Odor Data^3^, and the Food Flavor Lab^4^, with all odor threshold values based on water as the matrix.

To characterize the overall aroma profiles of the six Great Wall cigar types, the key aroma compounds were grouped into eight aroma categories based on similarities in their odor descriptors (Guo et al., 2021). Given the complexity of odor descriptors, some aroma compounds may exhibit multiple odor characteristics and therefore be assigned to more than one aroma category. For each aroma category, the rOAVs of all key aroma compounds assigned to that category were summed to obtain the total rOAV.

### 2.4 DNA extraction and amplicon sequencing

Total genomic DNA was extracted as described by Zhao et al. (2024). The V5–V7 region of the bacterial 16S rRNA gene was amplified using a semi-nested PCR approach. The primary amplification utilized the 799F/1392R primer pair, followed by secondary amplification with the 799F/1193R primer pair. For fungal community profiling, amplification of the ITS1 region was performed with the ITS1F/ITS2R primer pair. The nucleotide sequences of all primers and the corresponding PCR thermal cycling programs are summarized in Supplementary Table 1. Following library construction, the bacterial and fungal amplicon libraries were sequenced on an Illumina MiSeq PE300 platform. Further, bioinformatic processing of the raw sequencing reads was performed according to Yang et al. (2025a), including quality control, read merging, denoising, and amplicon sequence variant (ASV) inference. Taxonomic annotation was conducted using the SILVA database (v138.2) (Quast et al., 2013) for bacterial ASVs and the UNITE database (v9.0) (Abarenkov et al., 2024) for fungal ASVs.

### 2.5 Statistical analysis

R software (v4.4.2) and several online platforms were utilized for data analysis and visualization. Group differences were evaluated using one-way analysis of variance (ANOVA) and Duncan’s multiple range test (*p* < 0.05) with the agricolae package (v1.3.7). Microbial diversity indices were calculated using QIIME 2 on the Majorbio Cloud Platform (Ren et al., 2022). The same platform was also used to perform rarefaction curves, linear discriminant analysis effect size (LEfSe), UpSet plots, and principal coordinate analysis (PCoA). Principal component analysis (PCA) and partial least squares discriminant analysis (PLS-DA) were carried out on the Metware Cloud Platform^5^. The Spearman correlation heatmap was generated via the ComplexHeatmap package (v2.28.0).

## 3 Results

### 3.1 Microbial community composition across the six Great Wall cigar types

Based on 16S rRNA gene and ITS amplicon sequencing, all samples showed Good’s coverage values above 99.9%, and the rarefaction curves approached saturation as sequencing depth increased (Supplementary Figure 1). These results indicated that the sequencing effort was sufficient for microbial community profiling. The UpSet analysis of microbial ASVs among the six cigar types is shown in Supplementary Figure 2. The ASVs shared by all six cigar types accounted for only 1.18% of the total bacterial ASV pool (13 ASVs) and 3.85% of the total fungal ASV pool (21 ASVs). In contrast, unique ASVs constituted the majority of the observed ASV pool, indicating substantial heterogeneity in microbial composition among these cigar types. The microbial community composition was further compared at the phylum and genus levels (Supplementary Tables 2, 3). Across all cigar types, bacterial ASVs were assigned to six phyla and 96 genera, whereas fungal ASVs were classified into four phyla and 141 genera. In terms of bacterial phyla, Bacillota exhibited the highest relative abundance across the six cigar types (65.14% on average), followed by Actinomycetota (33.61% on average) (Figure 1A). Specifically, Bacillota was most abundant in S5 and least abundant in S3, whereas Actinomycetota showed the reverse pattern. The predominant bacterial genera across the six cigar types were *Staphylococcus*, *Corynebacterium*, *Aerococcus*, *Yaniella*, and *Atopostipes* (Figure 1B). Among these genera, *Staphylococcus* (53.99% on average) and *Corynebacterium* (29.25% on average) were the most abundant, with their combined relative abundance ranging from 78.56% to 95.32% across the six cigar types. In addition, *Aerococcus* was more abundant in S2 than in the other cigar types, whereas *Yaniella* and *Atopostipes* were more abundant in both S1 and S3. Compared with the bacterial communities, the distribution of fungal phyla was relatively consistent across the six cigar types (Figure 1C). Specifically, Ascomycota (92.04% on average) and Basidiomycota (7.92% on average) were the predominant phyla, together representing more than 99% of the fungal community. However, fungal community composition differed across the six cigar types at the genus level (Figure 1D). *Aspergillus* dominated the fungal communities of these cigar types, with its relative abundance ranging from 75.60% to 98.29% in S2–S6, whereas it was less abundant in S1 (48.07% on average). Additionally, *Stagonosporopsis* and *Sampaiozyma* showed the highest mean relative abundance in S1, accounting for 10.37% and 7.21%, respectively. By contrast, *Wallemia* and *Xeromyces* were the most abundant in S5, at 11.66% and 5.49%, respectively.

**FIGURE 1:**
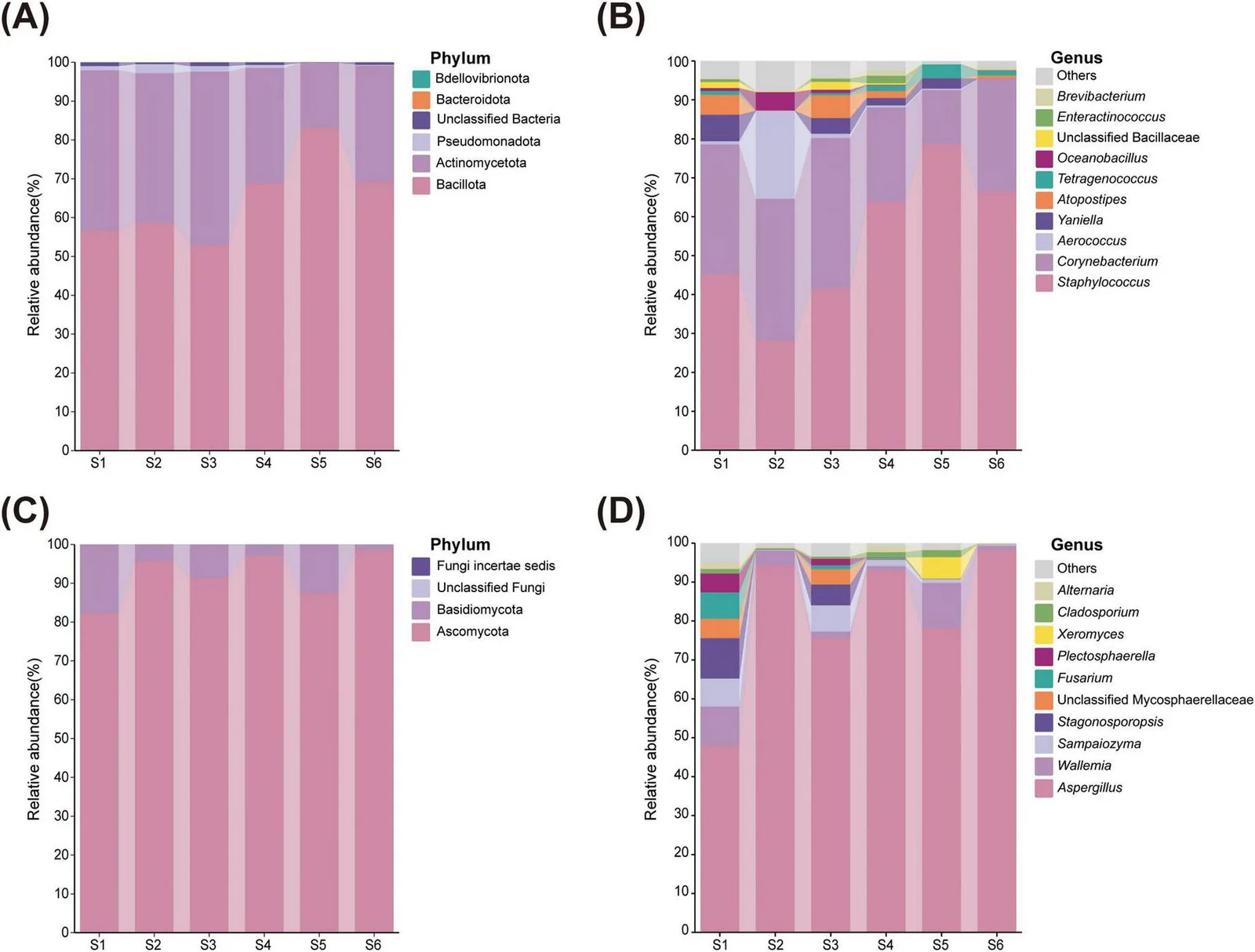
Microbial community structure across the six Great Wall cigar types. Relative abundance of bacterial communities at the phylum **(A)** and genus **(B)** levels. Relative abundance of fungal communities at the phylum **(C)** and genus **(D)** levels. S1–S6 represent the six cigar types: S1, Cameroon International; S2, Flourishing Age V; S3, Yichuan Laofang; S4, Miracle 132; S5, Spectacular No. 2; and S6, VegaFina Grand Corona.

### 3.2 Alpha and beta diversity of microbial communities across the six Great Wall cigar types

Significant differences in bacterial community richness and diversity were observed among the six Great Wall cigar types (Figure 2A). Specifically, S1–S3 showed higher bacterial community richness and diversity than S4–S6 (*p* < 0.05). Among the six cigar types, S3 exhibited the highest Chao1 index, suggesting the greatest bacterial richness, whereas S2 showed the highest Shannon index, suggesting the greatest bacterial diversity. Similarly, fungal alpha diversity indices differed significantly across the six cigar types (Figure 2B). Specifically, S1 showed the highest Chao1 and Shannon indices, followed by S3, suggesting that these two cigar types exhibited greater fungal richness and diversity than the other cigar types. It is noteworthy that S6 exhibited the highest Simpson index and the lowest Shannon index in both bacterial and fungal communities, indicating lower microbial diversity and a pronounced predominance of a few taxa. Furthermore, PCoA based on Bray–Curtis dissimilarities was utilized to evaluate the differences across the six cigar types (Figures 2C, D). The results demonstrated that the samples from different cigar types were distinctly separated in both bacterial and fungal communities. The first two principal coordinates collectively explained 91.43% and 93.68% of the total variation in bacterial and fungal community structure, respectively. Permutational multivariate analysis of variance (PERMANOVA) further revealed significant compositional differences among the six cigar types for both bacterial (R^2^ = 0.976, *p* = 0.001) and fungal (R^2^ = 0.962, *p* = 0.001) communities.

**FIGURE 2:**
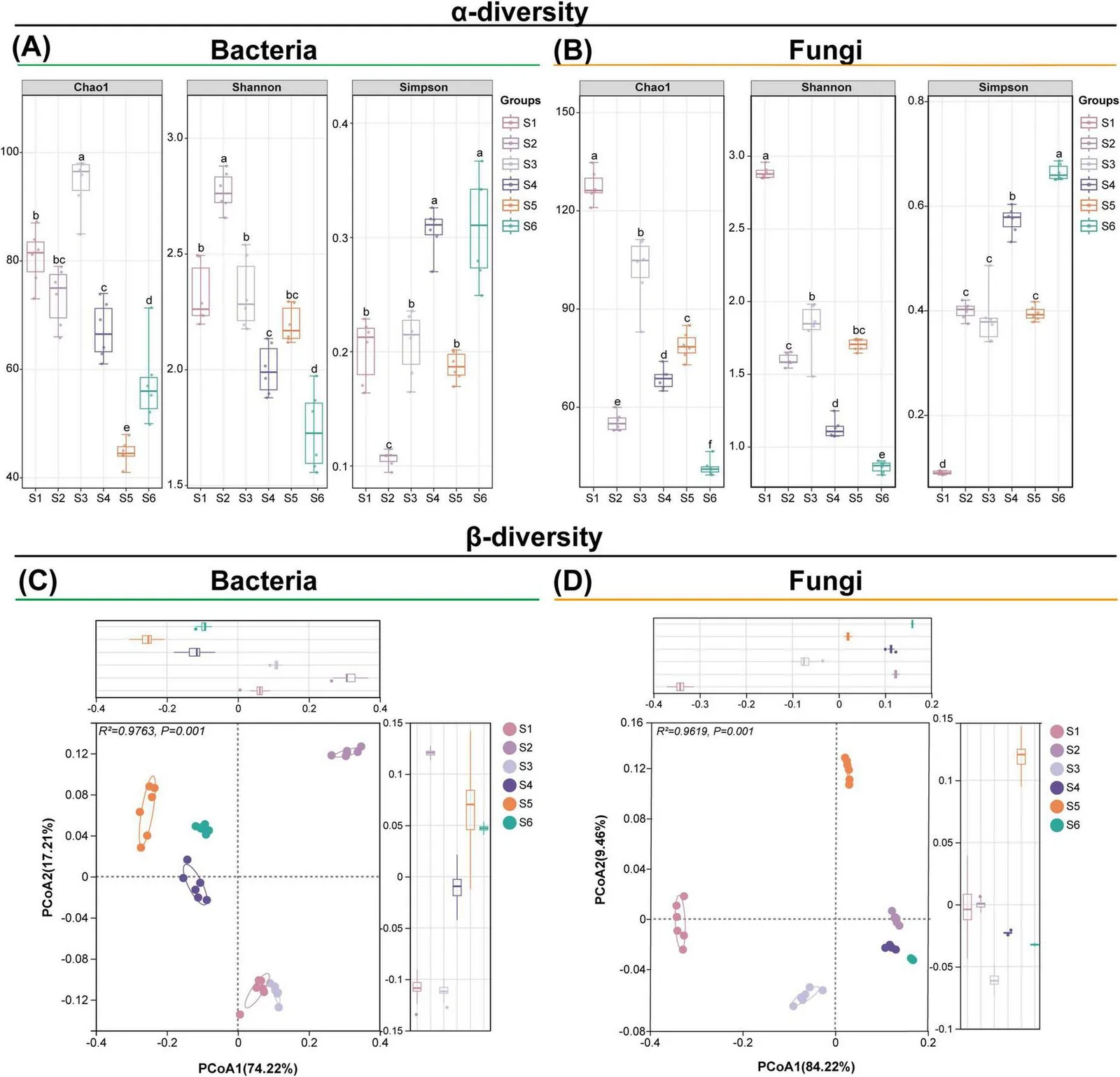
Microbial community diversity of the six Great Wall cigar types. Alpha diversity: **(A)** bacteria and **(B)** fungi; Beta diversity: **(C)** bacteria and **(D)** fungi. S1–S6 denote the six cigar types as defined in Figure 1. Different lowercase letters indicate significant differences in microbial diversity indices among the six cigar types (*p* < 0.05).

### 3.3 Screening for potential microbial biomarkers across the six Great Wall cigar types using LEfSe analysis

To further characterize taxonomic differences among the six cigar types, LEfSe analysis was conducted to identify differentially abundant taxa with an LDA score > 3.5 and *p* < 0.05. This analysis identified 16 differentially abundant bacterial genera, with 1, 4, 6, 2, 2, and 1 genera significantly enriched in cigar type S1 to S6, respectively (Supplementary Table 4). Similarly, 20 differentially abundant fungal genera were identified, with 12, 0, 2, 2, 3, and 1 genera significantly enriched in cigar type S1 to S6, respectively (Supplementary Table 5). To reduce stochastic noise associated with low-abundance genera, only genera with a mean relative abundance exceeding 0.5% were retained. Ultimately, 16 potential microbial biomarkers were selected, comprising nine bacterial genera and seven fungal genera. Specifically, *Stagonosporopsis*, *Sampaiozyma*, *Fusarium*, *Plectosphaerella*, unclassified Mycosphaerellaceae, and *Yaniella* were significantly enriched in S1; *Aerococcus* and *Pseudomonas* in S2; *Corynebacterium*, *Atopostipes*, and unclassified Bacillaceae in S3; *Enteractinococcus* in S4; *Wallemia*, *Staphylococcus*, and *Tetragenococcus* in S5; and *Aspergillus* in S6 (Figure 3). Collectively, these 16 genera were identified as potential microbial biomarkers for distinguishing among the six cigar types, providing a basis for subsequent exploration of microbe–VOC associations.

**FIGURE 3:**
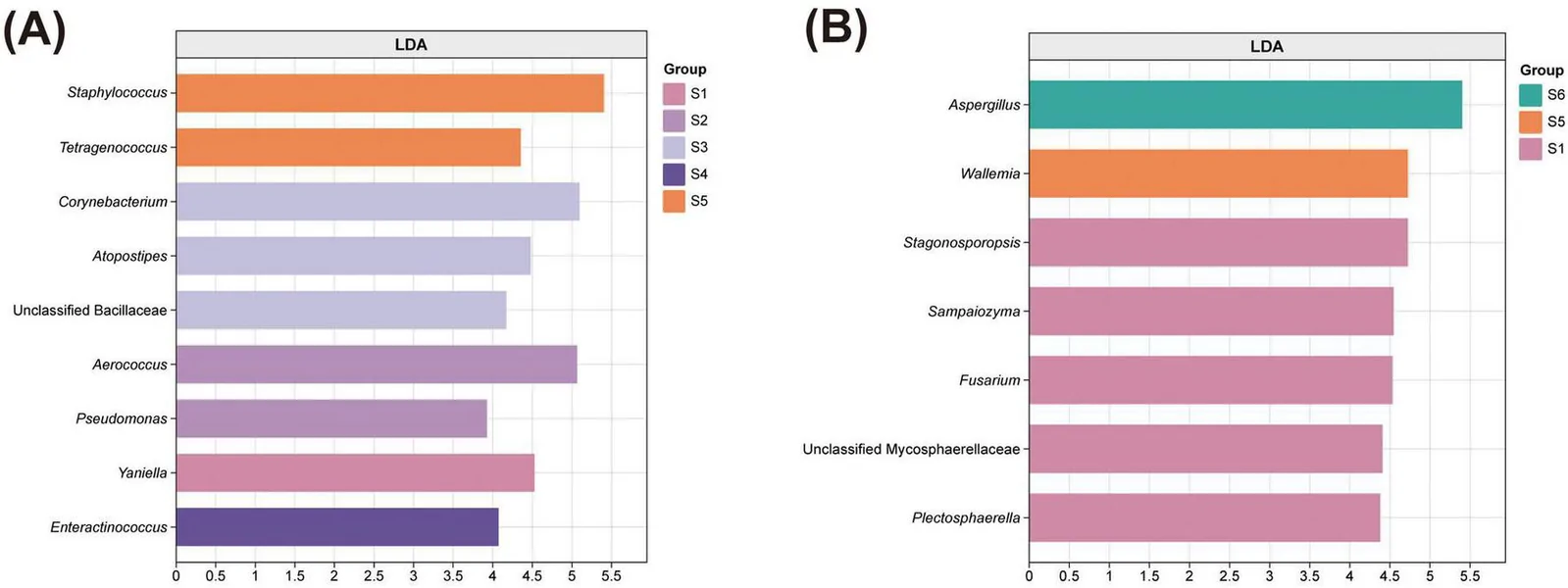
Potential bacterial **(A)** and fungal **(B)** biomarkers differentiating the six Great Wall cigar types. Biomarkers were identified via linear discriminant analysis effect size (LEfSe) analysis and mean relative abundance. S1–S6 denote the six cigar types as defined in Figure 1.

### 3.4 VOC profiles and compositional variation across the six Great Wall cigar types

Volatile metabolites in the six Great Wall cigar types were characterized using HS-SPME-GC-MS. VOC profiling identified 1,620 VOCs, which were assigned to nine chemical categories: terpenoids (349), esters (295), ketones (193), heterocyclic compounds (163), alcohols (116), aldehydes (85), acids (83), phenols (81), and other compounds (255) (Figure 4A and Supplementary Table 6). As shown in Figure 4B, the relative content of these chemical classes varied across cigar types. Nicotine and neophytadiene represented the most abundant individual VOCs, together contributing 37.43% of the total mean relative content. Across the six cigar types, both nicotine and neophytadiene showed higher relative content in S1 and S3, whereas their lowest relative content was observed in S2. At the chemical-class level, several classes showed significant differences across the six cigar types (*p* < 0.05). For example, S1 showed the highest relative content of terpenoids, esters, ketones, heterocyclic compounds, and aldehydes, whereas S5 had the highest relative content of alcohols and acids. To further evaluate the overall variation in VOC profiles among the six cigar types, PCA was performed using the relative content of the 1,620 VOCs. The PCA results indicated that the cumulative explained variance of the first two principal components was 44.85% (PC1 = 29.45%, PC2 = 15.40%). As illustrated by the PCA score plot (Figure 4C), QC samples were tightly clustered, demonstrating high analytical stability and reproducibility. Furthermore, biological replicates from the same cigar type formed compact clusters, whereas samples from different cigar types were clearly separated, suggesting substantial differences in VOC profiles across the six cigar types.

**FIGURE 4:**
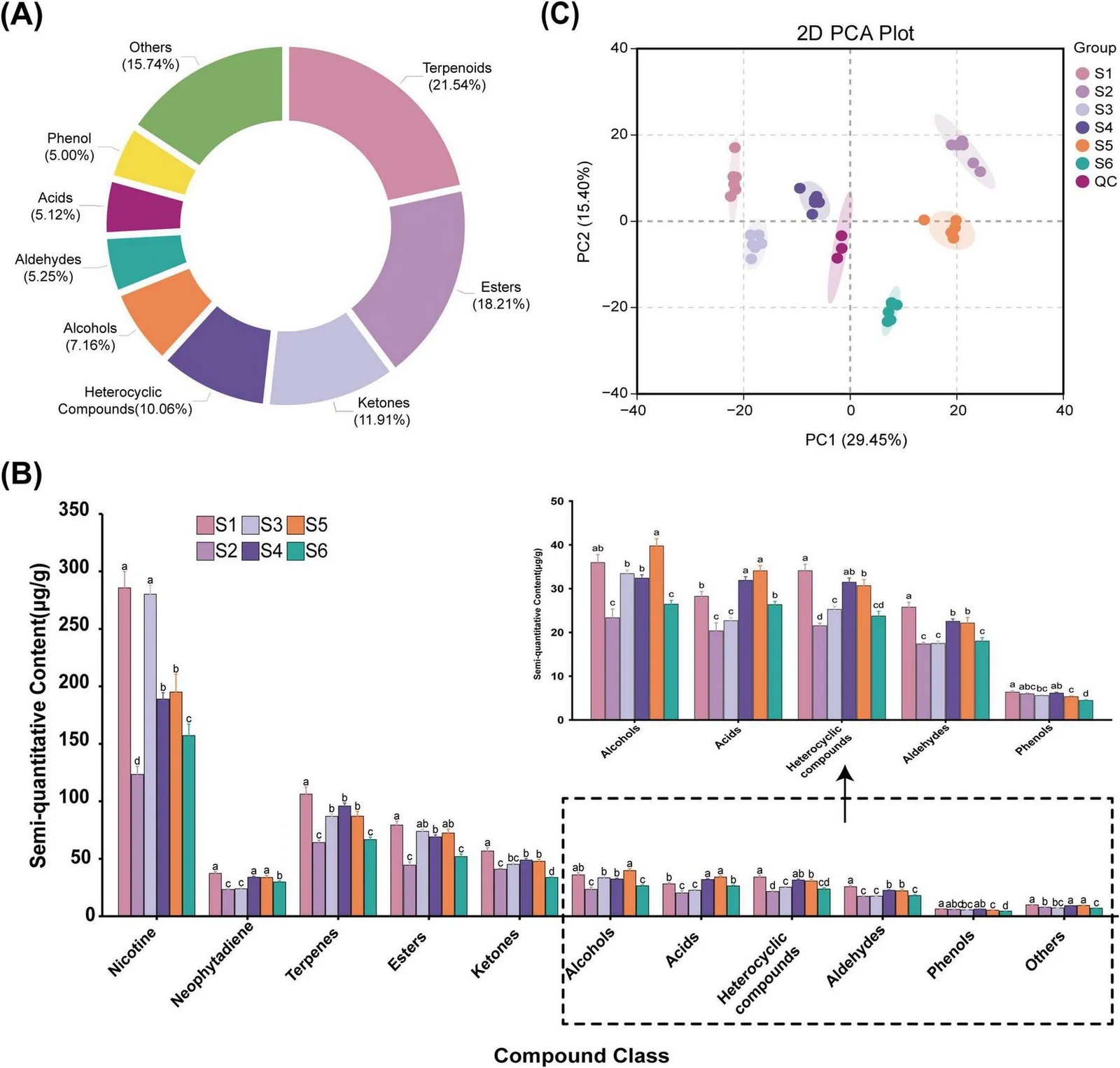
Classification and compositional profiles of volatile organic compounds (VOCs) among the six Great Wall cigar types. **(A)** Distribution of detected VOCs by chemical class. **(B)** Relative content of VOC classes among the six cigar types. Significant differences across the cigar types within the same VOC class are indicated by different lowercase letters (*p* < 0.05). **(C)** Principal component analysis (PCA) score plot of VOCs, with ellipses indicating 95% confidence intervals. S1–S6 denote the six cigar types as defined in Figure 1.

### 3.5 Identification of aroma-active compounds based on rOAV analysis

To screen the volatile compounds potentially contributing to cigar aroma profiles, rOAVs were calculated for all identified VOCs with available odor threshold data. The odor threshold values, odor descriptors, and corresponding rOAVs of these compounds across the six cigar types are provided in Supplementary Table 7. Based on the rOAV analysis, 48 VOCs with rOAVs exceeding 1 were regarded as aroma-active compounds. According to their chemical structures, these aroma-active compounds comprised 11 terpenoids, 10 ketones, eight esters, six aldehydes, five alcohols, three aromatic compounds, two heterocyclic compounds, one phenol, one ether, and one nitrogen-containing compound. Notably, 10 aroma-active compounds, represented by dihydro-2-methyl-3(2H)-furanone, phenylethyl isovalerate, and β-damascenone, exhibited rOAVs exceeding 100 in all cigar types.

### 3.6 Screening of key aroma compounds and aroma profiling across the six Great Wall cigar types

Partial least squares discriminant analysis was carried out using the relative content of compounds with rOAVs > 1 to screen for the key aroma compounds that contributed to discrimination among the six cigar types. The model demonstrated high explanatory and predictive performance, as indicated by R^2^*Y* = 0.980 and Q^2^ = 0.971. In addition, the 200-permutation test demonstrated that the Q^2^ intercept was negative, indicating the model was not overfitted (Supplementary Figures 3A, B). Variable importance in projection (VIP) scores are commonly used to identify key variables in PLS-DA, and variables with VIP > 1 are generally considered to contribute substantially to group discrimination. As shown in Supplementary Figure 3C, 17 key aroma compounds (VIP > 1, *p* < 0.05, and rOAV > 1) were identified, consisting of seven terpenoids, three ketones, three aldehydes, two esters, and two alcohols. Based on their odor descriptors, these 17 compounds were assigned to eight aroma categories: woody, sweet, roasted, nutty, fruity, floral, herbal, and creamy. To compare the aroma profiles among the six cigar types, the rOAVs of these compounds were Z-score normalized and visualized in a heatmap (Figure 5A). Furthermore, the total rOAV of these eight aroma categories across the six cigar types are illustrated in Figure 5B. The results showed that S1 and S3 exhibited higher rOAVs of phenylethyl isovalerate, phenylacetaldehyde, isomethyl-α-ionone, β-damascenone, 2-dodecanone, 3-acetylpyridine, and dihydro-2-methyl-3(2H)-furanone than the other four cigar types, thereby imparting stronger sweet, roasted, nutty, fruity, and floral notes. Among these seven compounds, dihydro-2-methyl-3(2H)-furanone and β-damascenone showed higher rOAVs than the other five compounds in S1 and S3 (Supplementary Table 7). By contrast, S4 and S6 exhibited higher rOAVs of helional, δ-cadinene, and β-farnesene, thus providing more pronounced woody and herbal notes. Notably, S2 and S5 showed stronger creamy notes, primarily due to the higher rOAV of δ-dodecalactone.

**FIGURE 5:**
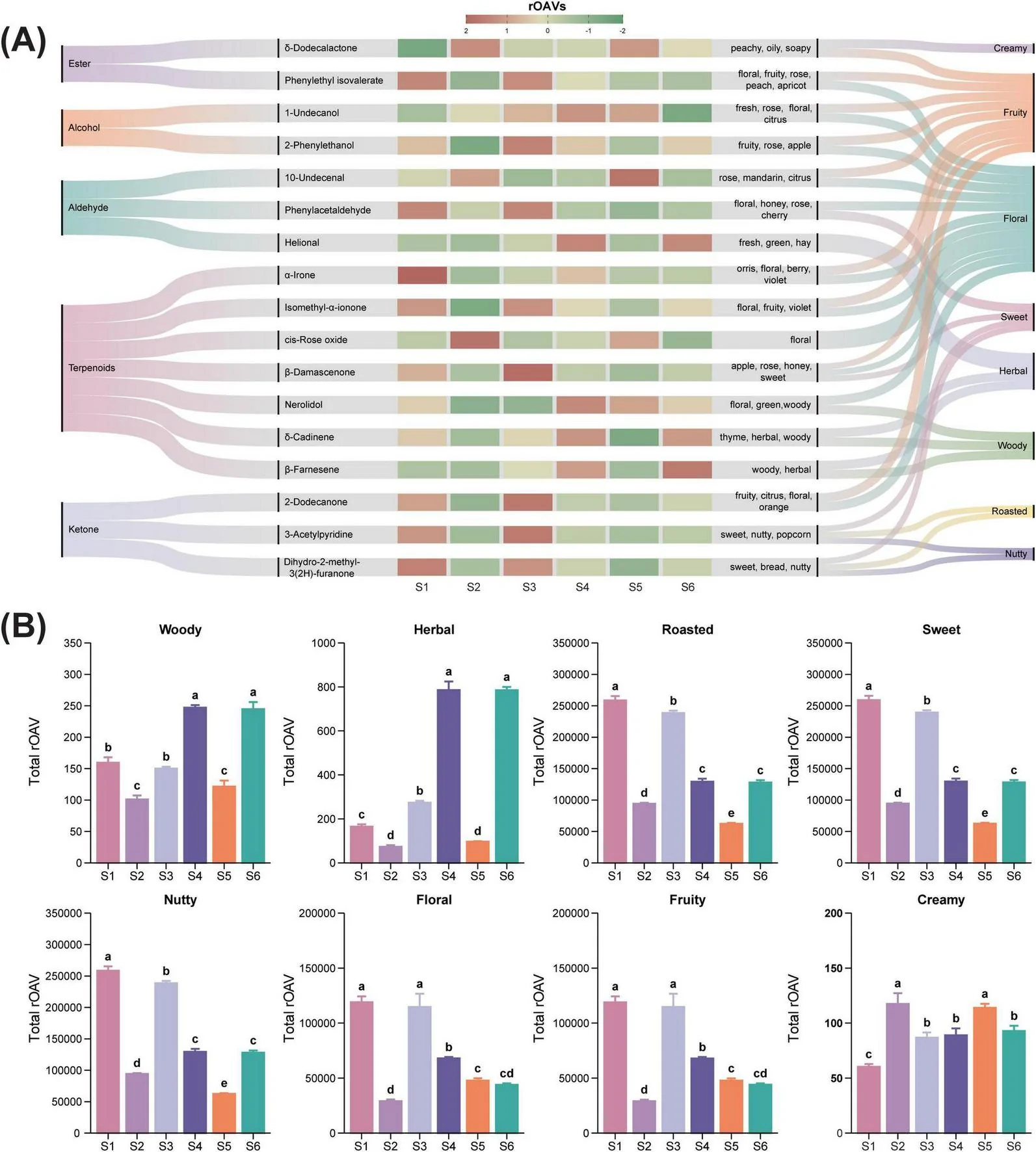
Aroma profiles and cumulative relative odor activity value (rOAV) contributions of key aroma compounds across the six Great Wall cigar types. **(A)** Chemical classes, rOAV distributions, aroma descriptors, and aroma categories of key aroma compounds. **(B)** Cumulative rOAVs of key aroma compounds assigned to each aroma category among the six cigar types. Significant differences across the cigar types are represented by different lowercase letters (*p* < 0.05). S1–S6 denote the six cigar types as defined in Figure 1.

### 3.7 Associations between potential microbial biomarkers and key aroma compounds

Potential microbe–VOC associations were explored by Spearman correlation analysis, and the false discovery rate (FDR) method was employed to adjust the *p*-values for multiple comparisons. In the bacterial community, unclassified Bacillaceae, *Atopostipes*, *Enteractinococcus*, and *Yaniella* exhibited similar correlation patterns with the key aroma compounds. These bacterial taxa exhibited significant positive correlations with 3-acetylpyridine, dihydro-2-methyl-3(2H)-furanone, and β-damascenone, but showed significant negative correlations with δ-dodecalactone (Figure 6A). In the fungal community, *Stagonosporopsis*, *Sampaiozyma*, *Fusarium*, unclassified Mycosphaerellaceae, and *Plectosphaerella* also displayed a comparable correlation pattern with the key aroma compounds. These fungal taxa displayed significant positive associations with 3-acetylpyridine, 2-phenylethanol, and phenylethyl isovalerate, but exhibited significant negative correlations with δ-dodecalactone (Figure 6B). In contrast to these fungal taxa, *Aspergillus* was significantly negatively correlated with the majority of the key aroma compounds, including 3-acetylpyridine, phenylethyl isovalerate, and 2-dodecanone.

**FIGURE 6:**
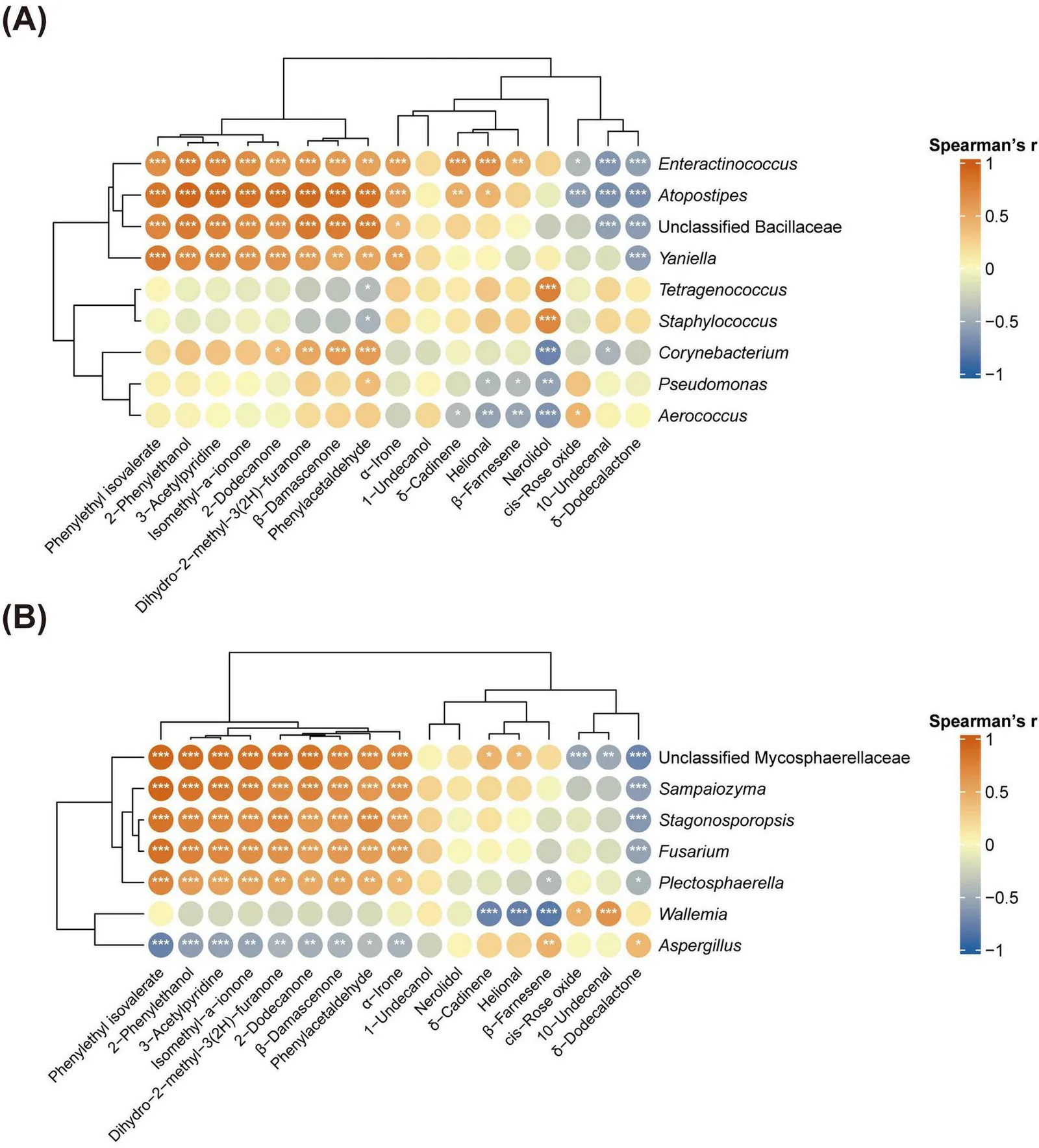
Spearman correlation heatmaps between key aroma compounds and potential microbial biomarkers. **(A)** Correlations between potential bacterial biomarkers and key aroma compounds. **(B)** Correlations between potential fungal biomarkers and key aroma compounds. *, **, and *** indicate *p* < 0.05, *p* < 0.01, and *p* < 0.001, respectively.

## 4 Discussion

Aroma is a key determinant of cigar quality, and it is largely shaped by the composition and relative content of VOCs. The generation and transformation of these volatile metabolites are closely linked to microbial community composition and microbial metabolic activities (Geng et al., 2025). Consequently, an integrated analysis of VOC profiles and microbial communities can elucidate aroma differences among different cigar types, thereby contributing to the refinement of cigar quality evaluation. In the present study, we systematically characterized the microbial community composition and VOC profiles of six Great Wall cigar types and investigated the associations between the potential microbial biomarkers and key aroma compounds. These findings offer valuable insights into how microbial composition contributes to aroma formation.

Microorganisms can participate in cigar aroma formation primarily through the enzymatic degradation of tobacco macromolecules and biotransformation of aroma precursors (Li et al., 2020). In this study, we employed 16S rRNA/ITS amplicon sequencing to characterize the microbial communities across the six cigar types. The dominant bacterial and fungal genera were generally similar across all samples, with *Staphylococcus* (Figure 1B) and *Aspergillus* (Figure 1D) predominating in the bacterial and fungal communities, respectively. This finding aligns with previous reports identifying these genera as major microbial taxa during air-curing and fermentation of cigar tobacco leaves (Li et al., 2024; Zhang et al., 2024a; Pei et al., 2025). *Staphylococcus* has been demonstrated to improve sensory quality by promoting the degradation of tobacco macromolecules, which may in turn facilitate the biosynthesis and enrichment of volatile aroma compounds (Ren et al., 2024). Similarly, *Aspergillus* can secrete various extracellular enzymes, including amylases and proteases, which may accelerate the hydrolysis of carbohydrates and proteins. These enzymatic processes may facilitate the transformation of aroma precursors into aroma compounds, thereby contributing to a desirable sensory quality of cigars (Li et al., 2024). However, *Aspergillus* has also been identified as a biomarker of moldy tobacco leaves, and excessive proliferation of this genus may contribute to mold growth, thereby impairing cigar sensory quality (Wu et al., 2024). Therefore, the higher relative abundance of *Aspergillus* observed in S2, S4, and S6 may suggest a greater risk of mold proliferation during subsequent storage. Thus, temperature and relative humidity should be carefully monitored and controlled to reduce this risk in these samples. Notably, *Stagonosporopsis* and *Xeromyces* were dominant in S1 and S5, respectively. Compared with well-documented genera such as *Aspergillus*, these genera have received relatively limited attention in previous studies of cigar products (Joshi et al., 2024). In addition, two unclassified taxa—unclassified Bacillaceae and unclassified Mycosphaerellaceae—were detected in the present study. However, the roles of these four microbial taxa in cigar processing and quality remain unclear and require further investigation. Furthermore, microbial diversity analysis (Figure 2) and LEfSe analysis (Figure 3) revealed that S1 generally exhibited relatively high microbial richness and diversity and harbored the largest number of potential microbial biomarkers. These differences may be attributable to variations in both the physicochemical properties of raw materials and specific processing parameters. On the one hand, cigar tobacco leaves from different geographical origins may harbor distinct initial microbiota and differ in chemical substrate profiles, which together may influence subsequent microbial succession (Wang et al., 2024; Geng et al., 2025). On the other hand, variations in temperature, relative humidity, and other processing parameters across the stages of air-curing, fermentation, and aging may also shape microbial succession and community structure (Xue et al., 2023; Gao et al., 2025).

The characteristic aroma of cigars primarily arises from the release of VOCs (Jiang et al., 2024). In this study, nicotine was the most abundant VOC across the six cigar types, followed by neophytadiene (Figure 4B), consistent with the findings reported by Zhang et al. (2024b). As the principal tobacco alkaloid associated with physiological strength, nicotine increases harshness and irritation when present at elevated levels (Patten and De Biasi, 2020). In contrast, neophytadiene, a degradation product of chlorophyll, has been reported to enhance smoke smoothness and reduce harshness (Hu et al., 2022). By combining PLS-DA with rOAV analysis, this study further identified 17 key aroma compounds that contributed substantially to the aroma differences across the six cigar types (Figure 5A). Among these key aroma compounds, several compounds, including isomethyl-α-ionone, δ-cadinene, and β-farnesene, have also been identified as key aroma compounds in previous research, supporting their consistent contributions to cigar aroma (Yang et al., 2025b). In contrast, some compounds such as 2-phenylethanol, phenylethyl isovalerate, and δ-dodecalactone have been detected in previous studies but were less frequently identified as key aroma compounds (Yang et al., 2025b). This discrepancy may be attributed to differences in sample characteristics and analytical methods (Zhou et al., 2024; Yang et al., 2025b; Zhu et al., 2026). The rOAV distribution patterns of these key compounds differed among the six cigar types, which may help explain their distinct aroma profiles (Figure 5B). Specifically, S1 and S3 exhibited more pronounced floral, sweet, fruity, roasted, and nutty notes than other cigar types. These distinctive aroma profiles were primarily owing to the relatively high rOAVs of several key aroma compounds, such as dihydro-2-methyl-3(2H)-furanone and β-damascenone. Dihydro-2-methyl-3(2H)-furanone is a typical Maillard reaction product associated with sweet, roasted, and nutty notes. Owing to its extremely low olfactory threshold, this compound may substantially contribute to the formation of the overall aroma profile even when present at low levels (Wu et al., 2023; Yang et al., 2025b). As a carotenoid degradation product, β-damascenone is typically characterized by floral, fruity, and honey-like notes, thereby enhancing the mellowness and smoothness of cigar smoke (Si et al., 2023). By contrast, stronger woody and herbal notes were observed in S4 and S6, which were attributed to the higher rOAVs of helional and δ-cadinene. Previous research has shown that helional gradually accumulates as cigar aging progresses, an increase that may be associated with a sensory shift from initial spicy pungency to milder woody attributes (Yang et al., 2025b). δ-Cadinene is a sesquiterpene biosynthesized from dimethylallyl diphosphate (DMAPP) and isopentenyl diphosphate (IPP). This compound imparts a typical woody aroma and has been detected at relatively high levels in high-quality cigars, suggesting a potential role in improving overall aroma harmony (Yang et al., 2024). In addition, S2 and S5 showed more pronounced creamy aroma notes, mainly related to the relatively higher rOAV of δ-dodecalactone in these two types. δ-Dodecalactone is a representative lactone characterized by fruity, creamy, and fatty notes, which together impart a well-rounded sensory profile to tobacco. It is commonly used as a flavoring ingredient in tobacco products to enhance sensory richness (Buettner-Schmidt et al., 2024).

Finally, the relationships between potential microbial biomarkers and key aroma compounds were examined using Spearman correlation analysis (Figure 6). Representative bacterial genera (*Atopostipes* and *Yaniella*) and fungal genera (*Sampaiozyma* and *Stagonosporopsis*) displayed similar correlation patterns with multiple key aroma compounds. These findings suggest that aroma differences across cigar types may be influenced by the collective contributions of microbial consortia rather than by a single microbial genus (Wang et al., 2024). Notably, *Atopostipes* and *Sampaiozyma* exhibited significant positive correlations with 11 and 9 key aroma compounds (predominantly terpenoids and ketones), respectively, making them the two genera most extensively associated with key aroma compounds in this study. *Sampaiozyma* has been reported as a predominant fungal genus in strong-aroma flue-cured tobacco and proposed to be involved in carotenoid degradation, potentially contributing to the formation of carotenoid-derived aroma compounds such as β-damascone and β-ionone (Li et al., 2026; Wang et al., 2024). In addition, *Atopostipes* has also been identified as a bacterial taxon for discriminating cigar tobacco leaves from different geographical regions and its relative abundance has shown positive association with nootkatone (Zheng et al., 2022). However, it is crucial to emphasize that correlation analysis alone cannot establish causal relationships between these microorganisms and the biosynthesis, release, or transformation of aroma compounds. Future studies should focus on combining stable isotope dilution analysis (SIDA), metagenomic analysis, and functional validation to more accurately quantify key aroma compounds and further elucidate the microbial functional genes and metabolic pathways potentially involved in cigar aroma formation.

## 5 Conclusion

In summary, this study characterized the microbial community composition and VOC profiles of the six Great Wall cigar types. Although *Staphylococcus* and *Aspergillus* were consistently the dominant bacterial and fungal genera, respectively, the microbial composition differed across the six cigar types. Volatile metabolomics analysis identified 17 key aroma compounds, and variation in their rOAV distribution accounted for aroma differences among the six cigar types. Furthermore, Spearman correlation analysis revealed that several potential microbial biomarkers—particularly *Atopostipes* and *Sampaiozyma* —displayed significant positive associations with diverse key aroma compounds, suggesting their contribution to cigar aroma formation. These findings may help explain the aroma differences among the six cigar types and offer valuable insights for selecting functional microorganisms to improve cigar quality.

## Data Availability

The datasets presented in this study can be found in online repositories. The names of the repository/repositories and accession number(s) can be found below: https://www.ncbi.nlm.nih.gov/, PRJNA1419536.

## Footnotes

1
http://www.thegoodscentscompany.com

2
http://perflavory.com

3
http://www.odour.org.uk/odour/index.html

4
http://foodflavorlab.cn/#/home

5
https://cloud.metware.cn/
