# Supplementary material for: Unraveling the microbiological and chemical basis of aroma differences in Great Wall cigars: insights from amplicon sequencing and volatile metabolomics
Source: Front Microbiol. 2026 Sep 25;17:1952964. doi: 10.3389/fmicb.2026.1952964 (PMC13649770; doi:10.3389/fmicb.2026.1952964)
Supplement: Supplementary file 1 [file Data_Sheet_1.zip › Supplementary file 1 .docx]

Supplementary Material

**1	Supplementary Figures**

**Supplementary Figure 1. Bacterial (A) and fungal (B) rarefaction curves across the six Great Wall cigar types.** **S1–S6 denote the six cigar types as defined in Figure 1.**

**Supplementary Figure 2. UpSet analysis of shared and unique bacterial (A) and fungal (B) ASVs across the six Great Wall cigar types. S1–S6 denote the six cigar types as defined in Figure 1.**

**Supplementary Figure 3: PLS-DA of VOC profiles among the six Great Wall cigar types. (A) PLS-DA score plot; ellipses denote 95% confidence ellipses. (B) Permutation test for model validation based on 200 random permutations. (C) VIP scores of key aroma compounds. S1–S6 denote the six cigar types as defined in Figure 1.**
